# Sizing up DNA nanostructure assembly with native mass spectrometry and ion mobility

**DOI:** 10.1038/s41467-022-31029-5

**Published:** 2022-06-24

**Authors:** Jeroen F. van Dyck, Jonathan R. Burns, Kyle I. P. Le Huray, Albert Konijnenberg, Stefan Howorka, Frank Sobott

**Affiliations:** 1 Biomolecular & Analytical Mass Spectrometry, Chemistry Department, University of Antwerp, Antwerpen, Belgium; 2 Department of Chemistry & Institute of Structural and Molecular Biology, University College London, London, UK; 3 School of Molecular and Cellular Biology & Astbury Centre for Structural Molecular Biology, University of Leeds, Leeds, UK; 4 Present Address: Thermo Fisher Scientific, Eindhoven, The Netherlands

**Keywords:** Supramolecular assembly, DNA, DNA nanostructures, Mass spectrometry

## Abstract

Recent interest in biological and synthetic DNA nanostructures has highlighted the need for methods to comprehensively characterize intermediates and end products of multimeric DNA assembly. Here we use native mass spectrometry in combination with ion mobility to determine the mass, charge state and collision cross section of noncovalent DNA assemblies, and thereby elucidate their structural composition, oligomeric state, overall size and shape. We showcase the approach with a prototypical six-subunit DNA nanostructure to reveal how its assembly is governed by the ionic strength of the buffer, as well as how the mass and mobility of heterogeneous species can be well resolved by careful tuning of instrumental parameters. We find that the assembly of the hexameric, barrel-shaped complex is guided by positive cooperativity, while previously undetected higher-order 12- and 18-mer assemblies are assigned to defined larger-diameter geometric structures. Guided by our insight, ion mobility-mass spectrometry is poised to make significant contributions to understanding the formation and structural diversity of natural and synthetic oligonucleotide assemblies relevant in science and technology.

## Abstract (web summary)

Interest in oligonucleotide nanostructures has recently surged in basic and applied research. Here, the authors use native mass spectrometry and ion mobility to elucidate a prototypical hexameric DNA barrel structure as well as intermediates and byproducts of the assembly reaction.

## Introduction

DNA and RNA can form remarkable three-dimensional structures relevant in biology and nanobiotechnology. Biological RNA assemblies are the functional core of transformative CRISPR^1,2^ and snRNP^3^, and in DNA nanotechnology nucleic acid assemblies can also be designed from scratch by taking advantage of the predictable Watson-Crick base pairing. Programming DNA sequences in engineered assemblies expands the range of structures beyond biology while delivering defined function as molecular scaffolds or molecular machines. The field of synthetic DNA structures is fueled by software advances to rationally design DNA origami^4,5^, the drastically reduced costs for synthesizing DNA, and the increasing range of applications in sensing, biocatalysis, and biomedicine^6–8^.

Determining the size, shape, and assembly pathway of the oligomeric nucleic acids is key for understanding their structure and improving their rational design. Current techniques including gel electrophoresis^9^, size exclusion chromatography^10,11^, and dynamic light scattering determine the nanostructures’ average size, while atomic force microscopy^4,12,13^ and electron microscopy^14–16^ visualize them with nanometer resolution. Yet, these methods do not give sufficient detail on the composition and stoichiometry of folding products, intermediates in the assembly pathway, or the homogeneity and stability of the nanostructures in different solvents and buffers.

**Fig. 1:**
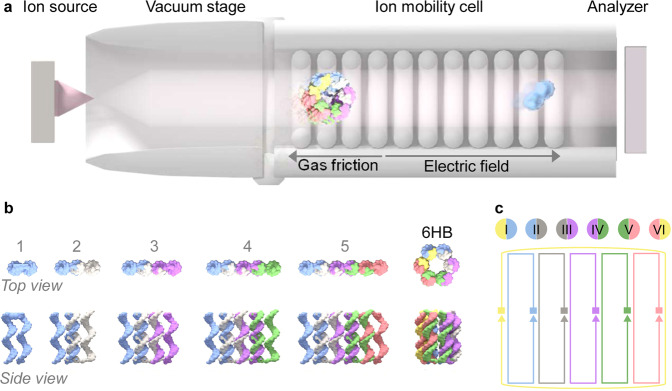
Native mass spectrometry and ion mobility analysis of DNA nanostructure assembly. **a** Schematic representation of the experimental setup for native nano-ESI IM-MS. **b** DNA helical barrel oligomerization from mono- (1) to pentamer (5) via pairwise double helix formation, and model of the hexameric helix bundle structure (6HB; individual strands are indicated in blue, gray, purple, green, red, and yellow). **c** Oligonucleotide connectivity map of individual DNA strands in the 6HB barrel (square indicates 5ʹ end and triangle 3ʹ).

Native mass spectrometry (MS) in combination with ion mobility (IM) has the potential to fill this gap in the analysis of DNA and RNA structures. Native MS can provide key insights into molecular details of subunit composition, stoichiometry, and stability of biomolecular assemblies, as demonstrated by the analysis of proteins^17–21^. Similarly, IM as a gas-phase electrophoretic technique (Fig. 1a) can determine the overall (i.e., rotationally averaged) size and shape of particles and is increasingly used to study protein conformations and complex topologies^22–25^. A unique strength of native IM-MS approaches is their ability to resolve heterogeneous distributions of dynamic non-covalent complexes and assembly intermediates, rather than just providing ensemble averages or resolving the most dominant species in these distributions^18,26^.

Applying IM-MS to investigate the assembly process of oligomeric DNA and RNA structures is, however, in its infancy. IM-MS has so far helped explore structurally simple short duplex DNA^27^, triple helices, and other prototypical motifs, such as DNA hairpins, pseudoknots, and cruciform structures^28–31^. Studies also measured the aptamer-ligand binding affinities^32^ to uncover how ligand binding strength relates to a concomitant structural reorganization^32,33^ and examined the dependence of folding and stability of biological G-quadruplexes on solution pH, counterions, and small molecule interactions^34–38^. These studies have improved the understanding of electrospray ionization (ESI) and different instrument-tuning parameters on the structure of oligonucleotides in gas-phase mass spectrometry^27,35,39^. Research addressing the use of native IM-MS for the characterization of topology and composition of larger, multi-subunit DNA nanostructures is however lacking to date^40,41^.

We set out to probe whether IM-MS can characterize the folding of DNA nanostructures and any assembly intermediates, to determine their stoichiometry, size, and shape. We employed an archetypal DNA nanostructure whose design is widely used across DNA nanotechnology. The structure is composed of six duplexes that are bundled together to form a six helix-bundle (6HB) barrel (Fig. 1b, c)^9,42^. The six duplexes are formed from six component oligonucleotides, numbered I to VI, where each strand is 50 bases long (Supplementary Table 1)^9^. Two adjacent oligonucleotides match uniquely and pairwise to form 21 base pair-long duplex regions (Fig. 1b, c). By analyzing the assembly of 6HB, we determine oligomeric species in the assembly pathway and separate them by mass and charge using native MS. Their shapes are probed with ion mobility by converting the recorded drift time into collision cross-section (CCS), which is a proxy for the overall size of the nanostructures.

## Results

### Characterization of DNA monomers

Before examining nanostructure assembly, we confirmed the sequence mass of the six individual component 50-mer DNA strands with native IM-MS in positive ionization mode. The experimentally determined mass of oligonucleotide I with 15,398.8 ± 0.3 Da matched well the calculated molecular weight of 15,397.98 Da with a phosphate on one and a free alcohol group on the other terminus (Supplementary Table 1). The other DNA strands showed similar spectra (Supplementary Fig. 1a) matching the calculated sequence masses (Supplementary Table 1) with the terminal phosphates present, except oligonucleotide VI where the observed mass corresponds to the lack of the terminal phosphate group (−79.98 Da).

The MS signals of the protonated oligonucleotides appeared predominantly in narrow charge state distributions with strong 7+ and 6+ peaks and a weak 5+ signal (Supplementary Fig. 1a). The charging is lower than the 8+ and 7+ charge states of similar-sized lysozyme protein under the same buffer and instrument tuning conditions. As charge states in electrospray ionization are known to correlate with the extent of exposed surface area^42^, our results indicate a single, relatively compact DNA structure.

Further detailed analysis of the MS signals revealed salt adducts and micro-heterogeneity of the DNA strands, depending on the MS ionization conditions. For example, oligonucleotide I’s mass/charge (*m*/*z*) range around the 6+ peak (Supplementary Fig. 1a) featured a few Na^+^ adducts which occurred due to the strong interaction of cations with the negatively charged DNA backbone, despite rigorous desalting procedures (see Experimental section). This behavior is typical for native mass spectra of oligonucleotides in the absence of Mg^2+^ ions as ubiquitous Na^+^ ions, for example from the borosilicate capillaries used for nano-ESI, readily take their place. Further investigation of the 6+ charge state of strand I showed minor signals corresponding to the lack of adenine and adenosine, most likely at the 5′ end of the sequence. Such micro-heterogeneities can be due to insufficient coupling efficiency in the synthesis, but could also result from excess energetic activation that some molecules might experience during MS analysis^43^.

To balance between effective declustering of analytes and avoiding their fragmentation and possible unfolding, we carefully tuned acceleration voltages in the interface of the mass spectrometer. As illustrated in Supplementary Fig. 2, increasing ion activation (voltage offsets: sampling cone, SC, and collision energy, CE) led to sharper and more symmetrical *m*/*z* peaks with reduced peak tailing. By comparison, the highest voltages yielded the best match to the calculated sequence mass due to complete declustering, yet at the price of minor peak shoulders due to low-level fragmentation. Ion mobility was also used to further evaluate the effect of acceleration voltages in the instrument. Excessive energetic activation would lead to unfolding that can be monitored by an increase in the collision cross-section. The CCS values extracted from the MS peaks (Supplementary Fig. 2, right column) depended on the precise *m*/*z* selection window, but as expected became slightly smaller as the extent of adducts was reduced. No unfolding was observed, indicating that the instrument tuning parameters were well optimized for this particular analyte, but the actual tuning conditions used in each case are reported in the corresponding figure legends. The accurate CCS value of 4457 ± 27 Å^2^ was obtained at the highest voltages from the narrow *m*/*z* range which matches the sequence mass (Supplementary Fig. 2c, red). Care should be taken with interpreting the CCS values as some oligonucleotides are known to adopt more compact structures in the gas phase than in solution^27^ (see below).

### Effect of ionic strength on nanostructure assembly

**Fig. 2:**
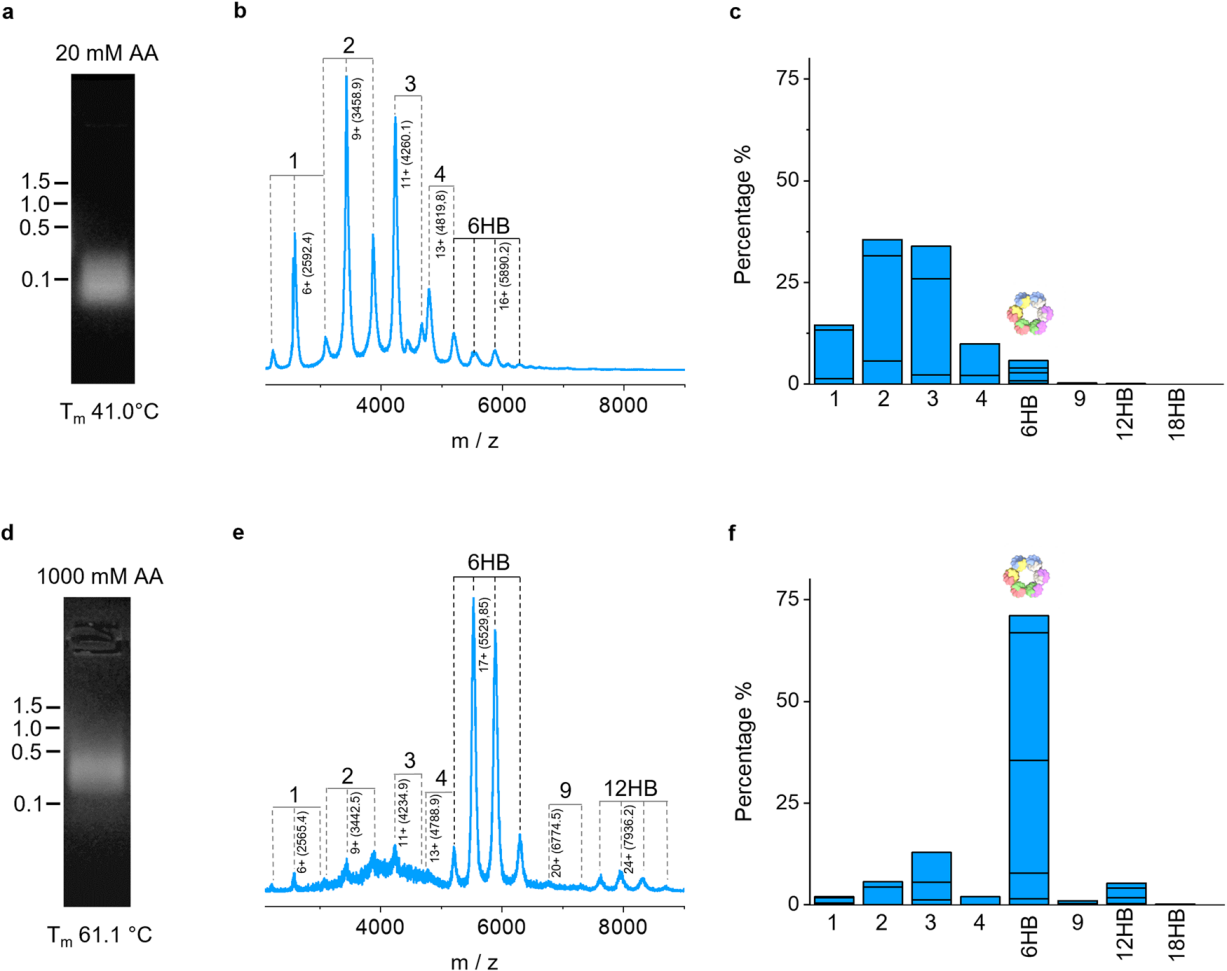
Effect of different ammonium acetate concentrations (20 and 1000 mM AA) on the formation of 6HB. **a**, **d** Native agarose gel. The numbers on the left indicate the molecular weight of the marker bands in kilo bases). **b**, **e** Native ESI-MS of an equimolar mixture of oligonucleotides I-VI after incubation shows the formation of hexameric 6HB but also the presence of other oligomeric species. Experimental parameters: sampling cone 125 V, trap collision energy 25 V, trap DC bias 50 V, trap pressure 4.38 × 10^−2^ mbar, and backing pressure 5.88 mbar. **c**, **f** Relative oligomer intensities at different AmAc concentrations; all species considered as indicated add up to 100% and segments in each bar indicate contributions from different charge states, with the highest at the bottom. Data for additional AmAc concentrations are shown in Supplementary Figs. 3 and 4.

To track the formation of the DNA nanostructure from component oligonucleotides, we first identified buffer conditions that support both molecular assembly and MS analysis. The assembly of DNA duplexes is usually enabled by cations including potassium^9^ that reduce repulsion between negatively charged DNA strands^44^. As KCl is not volatile and incompatible with native MS, possible alternatives include volatile bicarbonate or ammonium acetate (AmAc). Ammonium and acetate ions have ionic radii close to potassium and chloride, respectively. Here we investigated how DNA helix bundle assembly is influenced by ionic strength with AmAc concentrations ranging from 20 to 1000 mM. Using native gel electrophoresis as the first read-out method, 20 mM AmAc yielded smaller oligomer assemblies (Fig. 2a) while 1000 mM AmAc led to a higher assembly (Fig. 2d). The gel bands did not, however, provide accurate information on the oligomer composition and structure and did not offer any qualitative and quantitative information on assembly intermediates and progress.

Native MS provided unprecedented insight by identifying the type and relative amount of assembly intermediates, depending on the ionic buffer strength. Low salt strongly favored smaller oligomeric forms, particularly dimers (2), trimers (3), and tetramers (4) as evident in the native mass spectra (Fig. 2b and c). By comparison, high salt yielded the expected dominant hexameric nanostructure (6HB) with MS signals around *m*/*z* 6000 (Fig. 2e and f) and charge states ranging from 14+ to 18+ (Supplementary Figs. 3 and 4). Pentamers were conspicuously absent, which likely indicates positive cooperativity of binding the sixth strand to complete the 6HB structure. By surveying intermediate salt concentrations, we determined a threshold concentration of 200 mM AmAc above which the 91 kDa 6HB hexameric barrel structure became dominant; at a concentration greater than 700 mM it made up more than 70% of the total spectral intensity (Supplementary Figs. 3 and 4). As expected for the assembly promoting effect, increasing salt concentrations reduced the prevalence of smaller oligomers. Accompanying gel electrophoretic analysis confirmed the formation of a 6HB barrel band above 100 mM AmAc which co-migrated when folding was conducted in the standard buffer containing magnesium or potassium ions (Supplementary Figs. 5 and 6). Similar results on the equivalence of higher AmAc and magnesium and potassium salt concentrations were found when analyzing the stability of the structure via melting profiles (Supplementary Fig. 7).

We wondered, whether MS could also discern the composition of smaller assembly oligomers (Fig. 2) which must be heterogeneous in nature. For example, trimers can be made in six different ways, with three adjacent strands coming together such as 1-2-3, 2-3-4, 3-4-5, etc. (see connectivity scheme in Figs. 1b, c) with slightly different masses around ca. 3 × 15.4 = 46 kDa. Similar diversity is also expected for other oligomer sizes. Yet, the superposition of uncomplexed monomers under oligomerization conditions (Supplementary Fig. 1b) made it difficult to resolve the individual components fully and determine their relative intensities reliably. A compounding issue is the presence of Na^+^ adducts. Consequently, the overlapping higher-order oligomer peaks only allow for determining the average masses and oligomerization states (Supplementary Fig. 3). A more detailed analysis of the compositional heterogeneity of the intermediate species will require MS instrumentation capable of higher resolving power.

Using the existing instrumentation, the mass spectra pioneered the identification of larger nanostructures (Fig. 2) that were not detectable via gel electrophoresis^9^ (Supplementary Figs. 5 and 6). The larger nanostructures occurred above *m*/*z* 7000 as weak 9-mers, and, more prominently, as 12- (12HB, 188 kDa) and 18-mers (18HB, 277 kDa) (Fig. 2 and Supplementary Figs. 3 and 4). These larger structures with double and triple the hexamer weight could be representative of solution species but could also be an artifact of the electrospray process. For instance, several copies of the hexamer could end up in the same final droplet and form artificial multimers. Droplet-induced oligomerization has previously been observed for some proteins at high sample concentrations of 20 μM^45,46^. Yet, this oligomerization is unlikely at the low DNA concentration of 2 μM used in our study. To discard artefactual oligomerization due to (counter-)ion interactions, we acquired the spectra at opposite ionization polarity. Reassuringly, the 12HB assembly also occurred in negative ionization mode at similar charge states (Supplementary Fig. 8) albeit at a lower intensity. Our data showing the presence of large assembly products in the sample underscores the unique strength of mass spectrometric analysis to identify previously hidden DNA nanostructures.

### Ion mobility determines the size and shape of nanostructures

**Fig. 3:**
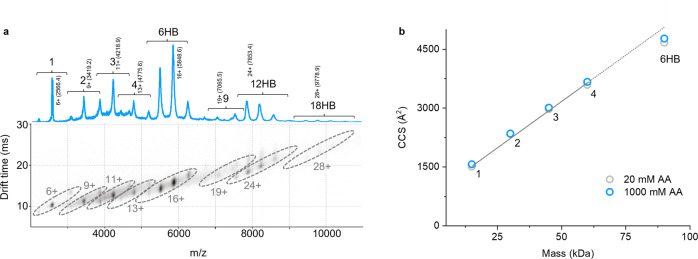
Ion mobility-mass spectrometry (IM-MS) analysis of nanostructure assembly at 300 mM AmAc. Experimental parameters: sampling cone 125 V, trap collision energy 25 V, trap DC bias 50 V, trap pressure 4.38 × 10^−2^ mbar, and backing pressure 5.88 mbar. **a** Top panel: native ESI-MS showing oligomers up to 18-mer (18HB). Bottom panel: corresponding drift time plot with different charge states (selected peaks annotated) of the same oligomer size grouped into ellipses. **b** Collision cross sections (CCS) derived from the measured IM drift times for different oligomer sizes after calibration, using the charge state with the highest peak intensity per group: (monomer (1) 6+, dimer (2) 9+, trimer (3) 11+, tetramer (4) 13+ and (6HB) 16+), at AmAc concentrations of 20 and 1000 mM. Monomer (1) to tetramer (4) shows a near-linear size increase, whilst the hexamer (6HB) is below the linear trend line (dotted), and therefore more compact. Note that the pentamer (5) was not detected.

With the oligomer distribution along the assembly pathway unraveled, we next aimed to identify the overall size and shape of the various assembly products. Ion mobility data associated with the spectra helped answer this question (Fig. 3, Supplementary Fig. 3). The drift times of the various assembly products are shown in a 2D plot, with different charge states of each oligomer grouped together in dashed ellipses (Fig. 3a). The individual ion mobility signals are well defined, suggesting that a single conformer or isomer dominates each charge state, rather than multiple coexisting states. Ion mobility also helps here to deconvolve overlapping signals, e.g., 8+ dimer (*m*/*z* 3865) and 12+ trimer (*m*/*z* 3861).

Using the drift times, we calculated the collision cross-section of the assembly products and thereby gauge their size. Strikingly, the CCS for oligomers increased linearly from mono- (1) to tetramers (4) with each additional subunit (Fig. 3b). This near linear relationship was independent of the buffer conditions as the CCS values were almost perfectly superimposed for the same species at 20 and 1000 mM AmAc. The linear trend indicates that each additional DNA strand leads to the elongation of a planar, sheet-like structure, similar to the previously described linear assembly of amyloid peptides leading to fibrils^47,48^. The CCS value obtained for the fully assembled 6HB barrel, however, lies below the linear trend (Fig. 3b), indicating that it adopts a more compact structure than a planar hexamer. This agrees with expectation as the additional sixth DNA strand interconnects the two ends of the planar “sheet” to roll it up into a barrel (Fig. 1b). Smaller oligomers (*n* < 6) do not have the ability to form closed cylinder-like shapes given the sequence design.

After demonstrating that ion mobility uncovers detailed conformational differences in the assembly products up to the 6HB barrel, we asked whether the analysis could also pinpoint the shape of the previously undetected “double” and “triple” species 12 and 18HB (Fig. 3a). The CCS values of these larger assemblies are not influenced by their charge state (Supplementary Fig. 9a and b), indicating a rather rigid structure that does not expand with increasing charge, as often observed for proteins. The sharp CCS distribution for the 12-mer (Fig. 3a) also suggests a particle with a well-defined shape and size.

To obtain more information on the overall architecture of the 12HB and 18HB assemblies, we compared the ion mobility data to calculated CCS values derived from structural models. CCS calculations of such oligonucleotide assemblies are not routine, particularly in the absence of suitable high-resolution data of similar structures which could be used for calibration, and algorithms not optimized for oligonucleotides. We used two software packages, IMPACT and IMoS, and applied different CCS calculation methods (PA, projection approximation; TJM, trajectory method; and EHSS, exact hard sphere scattering). Following this comprehensive analysis, the experimentally determined CCS value for the 6HB hexamer (4740 ± 40 Å^2^, Table 1) was found to match reasonably well with the calculated, rotationally averaged size of a barrel-shaped model using the IMPACT PA (4816 ± 18 Å^2^) or IMoS Monte Carlo PA (4865 Å^2^) methods without additional scaling. On the other hand, TJM results (obtained in IMPACT by recalibration of PA data using correlations derived from protein samples) and IMoS EHSS cross sections appear to overestimate the size of these DNA nanostructures considerably, based on these models. We decided to focus on IMPACT PA results in the following discussion.

**Table 1:** Experimental and calculated collision cross sections (Å^2^) representing the cylindrical 6-mer nanostructure (6HB) as well as suggested 12HB and 18HB models (Fig. 4).

		Calc. CCS (Å^2^)	Calc. CCS (Å^2^)	Calc. CCS (Å^2^)	Calc. CCS (Å^2^)
Oligomer	Exp. CCS (Å^2^)	PA IMPACT	PA (Monte Carlo) IMoS	TJM IMPACT	EHSS IMoS
Hexamer (6-mer)					
6HB	**4740** ± **40**				
Expected diameter barrel (6HB)		4816 ± 18	4865	6269 ± 25	6344
Squished barrel (6HB-SQ)		4624 ± 11			
Dodecamer (12-mer)					
12HB	**7545** ± **36**				
Linear side-by-side (12HB-a)		8174 ± 26	8286	10,932 ± 37	10,694
Stacked end-to-end (12HB-b)		8256 ± 29	8415	11,048 ± 41	10,750
Larger diameter barrel (12HB)		8364 ± 33	8471	11,200 ± 47	10,925
Squished barrel (12HB-SQ)		7374 ± 23			
Octadecamer (18-mer)					
18HB	**9578** ± **41**				
Triangle side-by-side (18HB-a)		10,647 ± 30	10,801	14,433 ± 43	14,321
Linear side by side (18HB-b)		11,530 ± 40	11,563	15,695 ± 58	14,953
Stacked end-to-end (18HB-c)		11,724 ± 44	11,889	15,972 ± 64	14,983
Larger diameter barrel (18HB)		13,364 ± 50	13,459	18,329 ± 72	17,236
Squished barrel (18HB-SQ)		10,879 ± 32			

Values in bold are the experimentally derived collision cross sections (CCS).

**Fig. 4:**
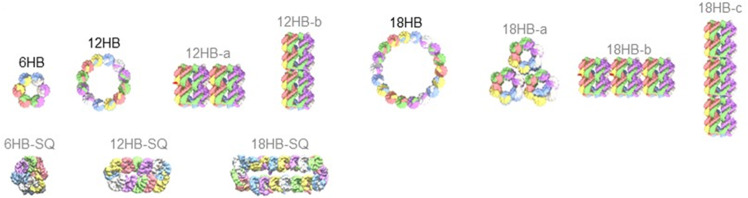
Representative models for the 6HB, 12HB, and 18HB structures used for calculating CCS values reported in Table 1. Multiple 6HB barrels are either placed side-by-side (12HB-a, 18HB-a, and 18HB-b) or stacked end- to- end (12HB-b and 18HB-c). The larger-diameter barrels 12HB and 18HB, next to 6HB, are also compacted laterally to generate the flattened and “squished” models 6HB-SQ, 12HB-SQ, and 18HB-SQ.

The experimental CCS values of the 12HB and 18HB are 7545 ± 36 Å^2^ and 9578 ± 41 Å^2^, which is 59% and 102% larger, respectively, than the 6HB (Table 1). In order to interpret the CCS values of the latter two oligomers, we initially considered various theoretical model structures including larger-diameter barrels such as 12HB or 18HB “sheets” rolled up to cylinders. Alternatives are stacks of multiple hexamers, either side-by-side parallel cylinders (12HB-a, 18HB-a/-b) or end-to-end elongated barrels (12HB-b and 18HB-c, see cartoons in Fig. 4). Only one of these models is expected to be correct, as our well-defined experimental CCS for the 12- and 18-mers rules out multiple coexisting structures with different CCS. It would appear that the best match for the 12-mer among the initial models has two 6HB cylinders attached side by side, with an IMPACT PA prediction of 8174 ± 26 Å^2^. Similarly, an arrangement of two 6HB stacked end to end represents an elongated cylinder with a slightly larger calculated CCS value. Analogous models for the 18-mer are composed of three 6HB. In contrast to these hypothetic and nonspecific assemblies, the design principle behind the formation of the 6HB allows for an oligomer size extension, with two or three sets of individual strands being able to form larger-diameter 12HB and 18HB barrels. Assuming perfectly circular cross-sections, these barrel structures are calculated to have the largest CCS of the models under consideration (Table 1; 8364 ± 33 Å^2^ and 13364 ± 50 Å^2^, respectively). They would also appear to be considerably larger than the experimental values (7545 ± 36 Å^2^ and 9578 ± 41 Å^2^, respectively), suggesting a mismatch with the expected structures.

We wondered if these findings could be due to structural changes of the DNA happening in the gas phase of the mass spectrometer. The study of biomolecular structure in the solution and gas phase is an active field of research, and both experimental and computational approaches have recently made significant progress in our understanding of the explicit role of solvation, and the consequences of its removal during the electrospray process^49,50^. Structures such as the large diameter barrels can obviously fluctuate and are in fact expected to undergo compaction in the gas phase due to the absence of water and the desire to self-solvate. The experimental CCS values are therefore expected to be smaller than those suggested by the naive models discussed above. For example, CCS analysis shows that DNA and RNA duplexes are compacted by up to 30% compared to canonical B helices^27^, whereas G-quadruplexes appear to be rather rigid under these conditions^51^. For our barrel structures, compaction could occur along the cylinder axis but also laterally to flatten the barrels, particularly the larger diameter ones. Since compaction along the axis should lead to similar (quasi-proportional) trends in CCS reduction for all models, we focused on lateral flattening and used Chimera software to build compacted (“squished”) models by displacing helices in the structures (Fig. 4, 6HB-SQ, 12HB-SQ, and 18HB-SQ). As expected, the 6HB appears quite rigid and the CCS of the squished model (6HB-SQ) is only reduced by ca. 4% (Table 1). Accordingly, the 12-mer and 18-mer models which consist of multiple 6HB barrels are equally unlikely to suffer significant size reduction. The larger-diameter barrels (12HB and 18HB) with their hollow structures can, by comparison, easily compact in the gas phase. We took the naive, cylindrical models and made geometric structures with similar cylinder axis lengths, but flattened them to achieve cigar-shaped cross-sections (with the round parts made up of 3 helices matching the curvature of the 6HB, Fig. 4) using Chimera in order to assess the effect which such compaction would have. The corresponding CCS PA values, calculated with IMPACT only, are close to the experiment (Table 1), suggesting that they are reasonable models for the experimental data. Based on these calculations, we suggest that the 12- and 18-mers represent larger-diameter barrels formed in solution which have undergone gas-phase compaction, rather than non-specific oligomers of the 6HB formed as ESI-MS clustering artifacts via droplet-induced aggregation.

## Discussion

In this report, we have utilized native IM-MS to investigate oligonucleotide self-assembly into larger functional DNA nanostructures, thereby addressing the demand for versatile tools to understand biologically and biotechnologically important DNA and RNA assemblies^52,53^. The IM-MS method can dissect dynamic and heterogeneous assembly products by mass and by size/shape, rather than just sampling average or dominant species of complex distributions. Both positive and negative electrospray ionization modes are suitable for non-denaturing analysis, but we found it easier to obtain good data in the positive mode. This is due to the ready availability of ion mobility calibration standards for complexes at high *m*/*z*, and the avoidance of Corona discharge effects which can occur with negatively charged nano-ESI capillaries, with detrimental effects on spectral quality.

Three highlights demonstrate the power of the analysis tool. First, MS identified previously undetected intermediates in the assembly process to the hexamer model structure. Pentamers were absent likely due to the positive cooperativity of binding the final strand to complete the hexameric barrel. The distribution of intermediates in the oligomerization process was strongly dependent on the ionic strength of the buffer solutions. Ammonium acetate provided sufficient ionic strength above 200 mM required for DNA annealing of the model 6HB barrel. Desalting of samples and careful tuning of the instrument was necessary to achieve declustering and good peak resolution. As the second highlight, the shape of the assembly products was tracked with ion mobility. The formation of a planar, sheet-like structure up to the tetramer was inferred from the linear trend in the collision cross-section upon subunit addition. By comparison, the more compact cylindrical barrel structure was successfully detected with the below-trend cross-section. Distinguishing between the shapes of the assembly intermediates is striking. As third highlight, MS detected previously unknown larger species with double and triple the mass of the 6HB barrel. By comparing the differences between the various structural models and our data, we concluded that these nanostructures are minor byproducts of the assembly process and most likely represent large diameter barrels that adopt flattened conformations in the gas phase.

To achieve these remarkable outcomes, several technical improvements had to be attained such as the use of a suitable volatile buffer to facilitate assembly and the fine-tuning of the MS ionization conditions which are particularly challenging for oligonucleotides since they are more prone to ionization-induced fragmentation than proteins. Recent developments in instrument and ESI source design will make it easier to identify even better ionization conditions in future experiments. Similarly, advanced molecular dynamics, CCS calculations, and other more refined computational tools are being adapted to further improve the characterization of the oligonucleotide structures.

We expect that the IM-MS method will prove highly beneficial in the widespread use of DNA and RNA for materials sciences as well as new drug delivery systems and vaccines. Powerful analytical approaches with high specificity and dynamic range are essential in this context. We anticipate that MS methods can address many challenges, both at the denaturing level for sequencing and mapping of modification types and sites, and natively for the investigation of assembly pathways and 3D structure. Approaches such as the ones presented here will likely make major contributions to our understanding of oligonucleotide nanostructures.

## Methods

### Reagents

All DNA oligonucleotides were purchased from IDT DNA technologies (Coralville, IA, USA) on a 250 nmol scale, with HPLC purification. All other reagents were purchased from Sigma-Merck (Gillingham, UK) unless stated.

### Sample preparation

The DNA constructs were assembled by combining equal volumes of oligonucleotides (Table S1)^9^ at 20 µM and diluted as required by the addition of ammonium acetate (AmAc) to generate a final concentration of 2 µM (each oligonucleotide). The aqueous AmAc solution (20–1000 mM) was adjusted to pH 8.5 with ammonium hydroxide solution. The constructs were folded by heating the solution to 95 °C for 2 min, followed by cooling to 4 °C at 1 °C min^-1^. The DNA samples were dialyzed additionally (Slide-A-Lyzer™ MINI Dialysis Device, 3.5 K MWCO, 0.1 mL (Thermo Fisher Scientific, Merelbeke, Belgium)) for a total of 24 h, with buffer renewal after 2, 4, 6, 8, and 10 h, against the corresponding AmAc concentration used during assembly (AmAc concentrations 20–1000 mM). Where required, samples were concentrated using 10 kDa molecular weight cut-off spin filters (Merck Millipore, Billerica, MA, USA) to the original starting volume.

### Nano-ESI ion mobility-mass spectrometry and data analysis

Nano-electrospray (ESI) IM-MS was performed using 2–4 μL of sample loaded into homemade gold-coated borosilicate glass capillaries, with spray voltages applied in the range 1.5–2.0 kV (positive) and 1.0–1.4 kV (negative mode). Spectra were recorded predominantly in positive mode, using an ion mobility enabled time-of-flight mass spectrometer (Synapt G2 HDMS, Waters, Wilmslow, UK). The following instrument parameters were carefully optimized for each sample, in order to avoid ion activation and preserve higher-order structure in the MS; their values are reported in the corresponding figure legends: sampling cone (SC), trap collision energy (CE) and trap DC bias voltage. Other important settings were: extraction cone: 2 V, transfer collision energy: 5 V, pressure in the source region (backing): 5.0–6.0 mbar, and in the trap cell (collision gas): 4.38 × 10^-2^ mbar.

IM-MS data were analyzed with Masslynx 4.1 and Driftscope 2.3 (both Waters, Wilmslow, UK). Collision cross-sections were calibrated using the lower positive charge states of calibrant proteins in the 18–336 kDa range, namely (β-Lactoglobulin (Bovine Milk), 18 and 36 kDa; BSA (Bovine Serum Albumin), 69 kDa; Concanavalin A (Canavalia Ensiformis), 103 kDa; Pyruvate Kinase (Rabbit Heart), 237 kDa, and Glutamate Dehydrogenase (Bovine Liver), 336 kDa) (all Sigma, St. Louis, MO, USA)^23^.

To determine oligomer abundances, we used ion mobility to separate peak series overlapping in *m*/*z*, and integrated their total intensity by area. Relative abundances are given as percentages of the total intensity of all species considered.

### Modeling and CCS calculations

In order to generate DNA nanostructure models, double helices formed by pairing of monomer DNA strands were produced with Web 3DNA 2.0 software^54,55^. A hexameric barrel was built by manual docking of individual monomers and connecting strands consisting of 4 thymidines were added in the Yasara Dynamics software suite (Version 16.4.6)^56^. After each additional double helix, energy minimization was performed using the integrated algorithm in the Yasara program. This was also done for the larger diameter 12HB and 18HB models, while the alternative structures were produced from stacked 6HB dimers or trimers. The final models were equilibrated following a 3 ns molecular dynamics run using Amber99^57^ to remove steric clashes and allow backbone relaxation.

Gas-phase compaction of 6HB, 12HB, and 18HB structures was modeled using UCSF Chimera developed by the Resource for Biocomputing, Visualization, and Informatics at the University of California^58^. Individual helices were laterally displaced in these models, while maintaining the overall cohesion of the structures.

CCS values for the different models were calculated with IMPACT software^59^ which uses the projection approximation (PA) method and also provides estimated trajectory method (TJM) data by a recalibration procedure. The IMoS software suite (v1.04b, http://www.imospedia.com/) was also used with the Monte Carlo projection approximation (PA) and exact hard sphere scattering (EHSS) methods, without correction factor and using N_2_ as drift gas. A comparison of the different CCS calculation methods, and the typical errors associated with their application to protein structures, is provided in ref. ^59^.

### Agarose and polyacrylamide gel electrophoresis

The DNA constructs were assembled by mixing equimolar amounts of component DNA strands (0.5 µM) containing the stated amount of AmAc (20–1000 mM), magnesium chloride (2–16 mM), or potassium chloride (100–1000 mM) containing 1x TAE pH 8.3. The DNA assemblies were folded by heating the solution from 95 °C for 2 min, and cooling to 20 °C at a rate of 5 °C per min.

The assembled DNA barrels were analyzed using 1.3% agarose gel using tris-acetate-EDTA (TAE) buffer pH 8.3. A solution containing 5 pmol DNA was mixed with 5 μL gel loading dye before transferring the solution into wells. The gel was run at 60 V for 60 min at 8 °C. The bands were visualized by UV illumination after staining with ethidium bromide solution. A 100 bp marker (New England Biolabs, Hitchin, UK) was used as the reference standard.

For polyacrylamide gel electrophoresis (PAGE), the assembled DNA barrels were analyzed using a 10% polyacrylamide gel with a 5% stack using 1x TAE running buffer pH 8.3. A solution containing 6–10 pmol DNA was mixed with 6 μL gel loading dye before loading into the wells. The gel was run at 160 V for 60 min at 8 °C. The bands were visualized by staining with ethidium bromide solution followed by UV illumination. A 100 bp marker (New England Biolabs, Hitchin, UK) was used as the reference standard for migration^9^.

### UV melting profiles

Thermal melting studies were performed using a UV-vis spectrophotometer (Varian Cary Eclipse, Agilent, UK) by monitoring the absorption at 260 nm of DNA constructs (0.1 µM, 150 µL, in the stated media type) in a 1 cm quartz cuvette. Heating and annealing were performed in 1 °C steps for 1 min between 85 and 20 °C.

### Reporting summary

Further information on research design is available in the Nature Research Reporting Summary linked to this article.

## Supplementary information

Supplementary Information

Reporting Summary

## Data Availability

All data and models generated in this study have been deposited at 10.5518/1153.
