# Supplementary material for: Sizing up DNA nanostructure assembly with native mass spectrometry and ion mobility
Source: Nat Commun. 2022 Jun 24;13:3610. doi: 10.1038/s41467-022-31029-5 (PMC9232653; doi:10.1038/s41467-022-31029-5)
Supplement: Supplementary file 1 — Supplementary Information [file 41467_2022_31029_MOESM1_ESM.docx]

**Supplementary Information:**

**Sizing up DNA nanostructure assembly with
native mass spectrometry and ion mobility**

*Jeroen F. van Dyck^1ǂ^, Jonathan R. Burns^2ǂ^, Kyle I. P. Le Huray^3^, Albert Konijnenberg^1,4^,
Stefan Howorka^2*^, Frank Sobott^1,3*^*

^1^ Biomolecular & Analytical Mass Spectrometry, Chemistry Department, University of Antwerp, Antwerpen, Belgium

^2^ Department of Chemistry & Institute of Structural and Molecular Biology, University College London, London, UK

^3^ School of Molecular and Cellular Biology & Astbury Centre for Structural Molecular Biology, University of Leeds, Leeds, UK

^4^ Current address: Thermo Fisher Scientific, 5651 CA Eindhoven, The Netherlands

*^ǂ^These authors contributed equally*

**These authors jointly supervised this work*

*Corresponding authors: Frank Sobott, f.sobott@leeds.ac.uk; Stefan Howorka, s.howorka@ucl.ac.uk*

**Supplementary figures**

| ID | Sequence 5' 🡪 3' | Calc. MW (Da) | Exp. MW (Da) |
| --- | --- | --- | --- |
| I | **AGCGAACGTGGATTTTGTCCGACATCGGCAAGCTCCCTTTTTCGACTATT** | 15,397.98 | 15,398.8 ± 0.3 |
| II | **CCGATGTCGGACTTTTACACGATCTTCGCCTGCTGGGTTTTGGGAGCTTG** | 15,476.99 | 15,477.6 ± 0.3 |
| III | **CGAAGATCGTGTTTTTCCACAGTTGATTGCCCTTCACTTTTCCCAGCAGG** | 15,348.94 | 15,349.3 ± 0.2 |
| IV | **AATCAACTGTGGTTTTTCTCACTGGTGATTAGAATGCTTTTGTGAAGGGC** | 15,547.09 | 15,547.9 ± 0.2 |
| V | **TCACCAGTGAGATTTTTGTCGTACCAGGTGCATGGATTTTTGCATTCTAA** | 15,467.04 | 15,468.0 ± 0.3 |
| VI | **CCTGGTACGACATTTTTCCACGTTCGCTAATAGTCGATTTTATCCATGCA** | 15,331.95 | 15,252.7 ± 0.6 |

**Supplementary Table 1.** Name (ID), sequence, calculated and experimentally determined molecular weight (MW) of DNA 50-mer strands used to prepare DNA nanostructures.

**Supplementary Figure 1**. Native ESI-MS analysis of individual 50-mer DNA strands I - VI (2 µM in 500 mM AmAc). **a**) Spectra of each monomer, with insets showing the 6+ charge state with Na^+^ adducts and indicating mass losses. **b**) Superposition of 6+ charge states of all individual DNA strands I - VI from **a**. The intensity scale was kept fixed. The grey line shows the actual monomer distribution in the corresponding m/z range from Supplemental Figure 3 (the 200 mM spectrum was used but other AmAc concentrations look similar). Experimental parameters: sampling cone, 90 V; trap collision energy, 30 V; trap DC bias, 45 V; backing pressure, 5.5 mbar; trap pressure, 4.0·10^-2^ mbar.

**Supplementary Figure 2.** Desolvation and fragmentation of DNA. Native ESI-MS analysis in positive mode of the 16+ charge state of the assembled hexamer (500 mM AmAc) at different declustering voltages leading to **a**) low, **b**) medium, and **c**) high activation. The values for the sampling cone (SC) voltage and trap collision energy (CE) are indicated. The panels on the right show drift time profiles corresponding to an identical, fixed m/z range (red lines), and an m/z window of varying width around the peak apex (black lines). **c**) Effect of collisional activation observed with the stepwise loss of mass (ca. 162 ± 20 Da; magenta and green dotted lines).

**Supplementary Figure 3**. Native ESI-MS analysis of the 6HB and larger assemblies as well as remaining smaller oligomers measured at different ionic strengths, with AmAc concentrations ranging from 20 to 1000 mM. The intensity scale was fixed across spectra; oligomer labels are the same as in Figure 2. The most abundant observed charge state is labeled for each oligomer. Experimental parameters: sampling cone, 125 V; trap collision energy, 25 V; trap DC bias, 50 V; backing pressure, 6.0 mbar; trap pressure, 4.4·10^-2^ mbar. The DNA strand concentration was 2 µM per monomer.

**Supplementary Figure 4**. Relative oligomer intensities at different AmAc concentrations derived from data shown in Supplementary Figure 3. All indicated oligomer classes add up to 100% and segments in each bar indicate contributions from different charge states, with the highest charge state at the bottom.

**Supplementary Figure 5**. Native 2% agarose gel electrophoretic analysis of DNA 6HB formed under different salt conditions. The DNA nanostructure was assembled at a concentration of 1 µM in either ammonium acetate (1000-20 mM), in magnesium chloride (2-16 mM) or potassium chloride (100-1000 mM). M denotes the 100 bp DNA-ladder marker; the numbers at the left indicate the DNA lengths in bp. The data indicate that 6HB assembly occurred above 2 mM Mg^2+^, and above 100 mM K^+^ or NH_4_^+^.

**Supplementary Figure 6**. Native 10% polyacrylamide gel electrophoretic analysis of DNA 6HB formed under different salt conditions. The DNA nanostructure was assembled at a concentration of 1 µM in either ammonium acetate (1000-20 mM), in magnesium chloride (2-16 mM) or potassium chloride (100-300 mM). M denotes the 100 bp DNA-ladder marker; the numbers at the left indicate the DNA lengths in bp. The data indicate that 6HB assembly occurred above 100 mM K^+^ or NH_4_^+^.

**Supplementary Figure 7**. UV melting profile analysis of DNA 6HB in **a**) ammonium acetate and **b**) magnesium chloride. The analysis was conducted at a DNA nanostructure concentration of 0.1 µM by monitoring the absorbance at 260 nm at a heating rate of 1 °C/min.

**Supplementary Figure 8.** Native MS analysis of DNA nanostructures measured in positive and negative mode (both 2 µM in 500 mM AmAc). Charge states are similar in both ionization modes and the most abundant hexamer (6HB) peaks are 17+ and 16-.

**Supplementary Figure 9.** Experimental and theoretical CCS values of 6HB (91 kDa), 12HB (182 kDa) and 18HB (273 kDa). All observed charge states (x axis) are shown with the dotted lines representing the theoretical CCS values (Å^2^) for the barrel-shaped 6HB and alternative 12HB and 18HB model structures (see Table 1 and Figure 4) Theoretical CCS values were calculated using the PA method in IMPACT.
